# Supplementary material for: Rare variants and founder effect in the Beauce region of Quebec
Source: Commun Biol. 2025 Aug 8;8:1184. doi: 10.1038/s42003-025-08630-7 (PMC12334588; doi:10.1038/s42003-025-08630-7)
Supplement: Supplementary file 1 — Supplementary Material [file 42003_2025_8630_MOESM1_ESM.pdf]

# Rare variants and founder effect in the Beauce region of Quebec

**Mylène Gagnon[1,2], Claudia Moreau[1,2], Jasmin Ricard[3], Marie-Claude Boisvert[3], Alexandre Bureau[3,4], Michel Maziade[3,5], Simon L. Girard[*1, 2, 3, 5]**

[1]Département des sciences fondamentales, Université du Québec à Chicoutimi, Saguenay, Québec, Canada.

[2]Centre Intersectoriel en Santé Durable, Université du Québec à Chicoutimi, Saguenay, Québec, Canada.

[3]Centre de recherche CERVO, Université Laval, Québec, Québec, Canada.

[4]Department of Social and Preventive Medicine, Faculty of Medicine, Université Laval, Québec, Québec, Canada

[5]Department of Psychiatry and Neuroscience, Faculty of Medicine, Université Laval, Québec, Québec, Canada

[6]Projet BALSAC, Université du Québec à Chicoutimi, Saguenay, Québec, Canada

***Corresponding author:**
Simon L. Girard
simon2_girard@uqac.ca

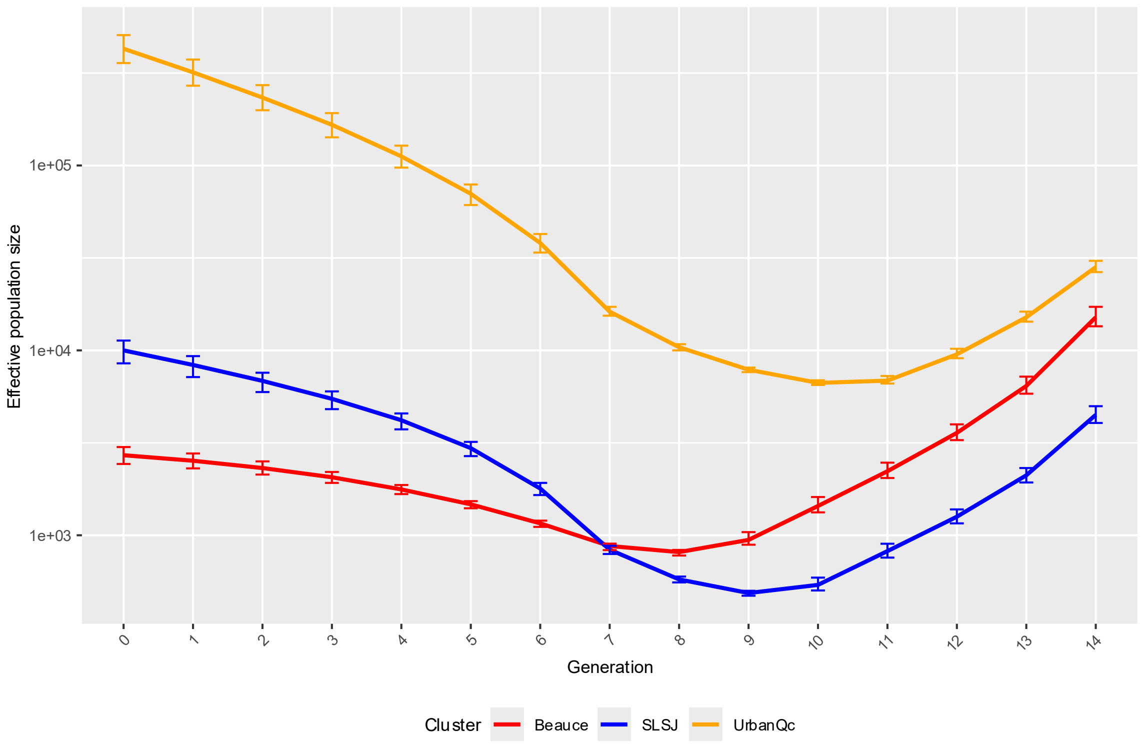


**Supplementary Figure S1.** Effective population size inferred on the genotyping data for the Beauce, SLSJ and UrbanQc clusters. Error bars are 95% bootstrap confidence intervals. The y axis is plotted on a log scale. Effective population size was determined using the ibdne software (version 23Apr20.ae9) with default parameters. (Red: Beauce cluster, Orange: UrbanQc cluster, Blue: Saguenay–Lac-St-Jean (SLSJ) cluster)

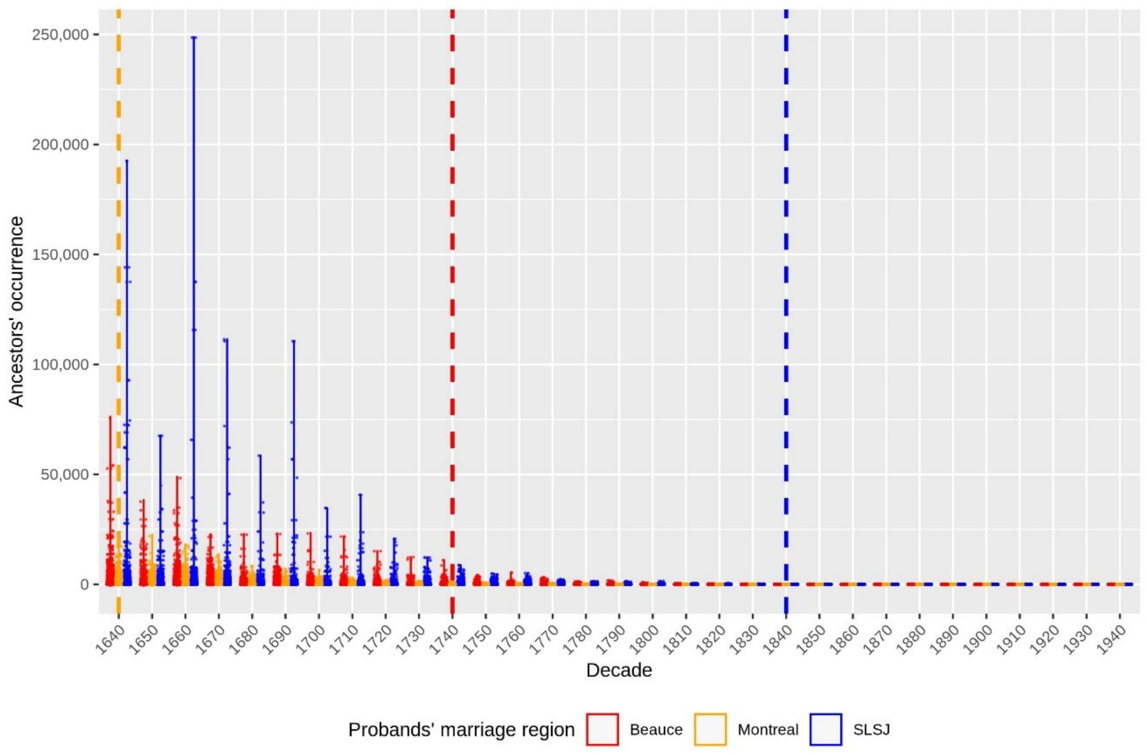


**Supplementary Figure S2.** Violin plot of the number of occurrences per ancestor per decade for each regional group. Occurrence refers to the number of times an ancestor appears across all genealogies, including repeated appearances within a single genealogy. Vertical dotted lines represent the decade of the beginning of the settlement for each region (Red: Beauce, Orange: Montreal, Blue: Saguenay–Lac-St-Jean (SLSJ**)).**

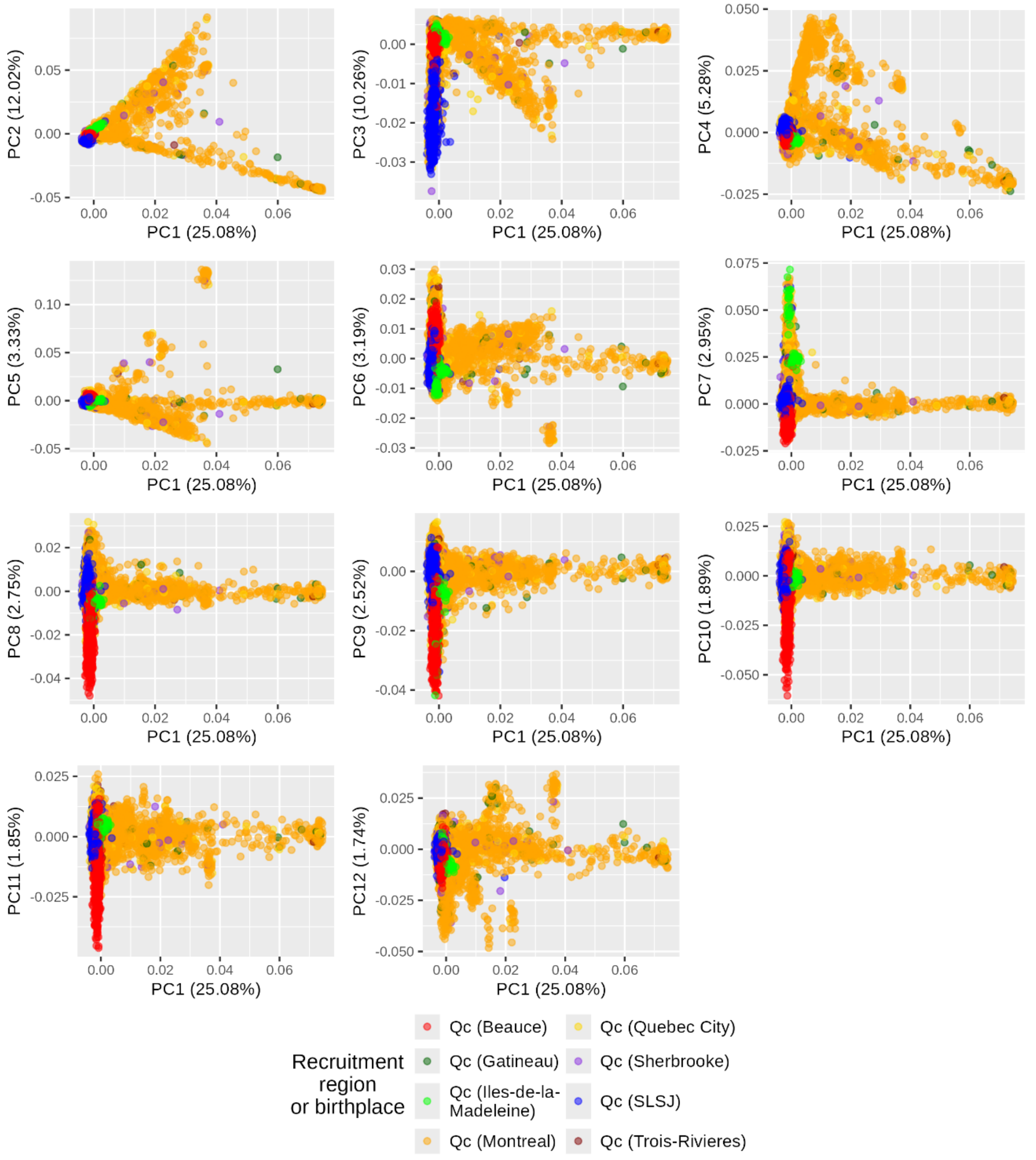


**Supplementary Figure S3.** Principal component analysis of the genotyping data using the PC-AiR method to account for relatedness (proportion of total IBD sharing < 0.125 or genealogical kinship < 0.0625). Individuals are colored by recruitment region or birthplace.

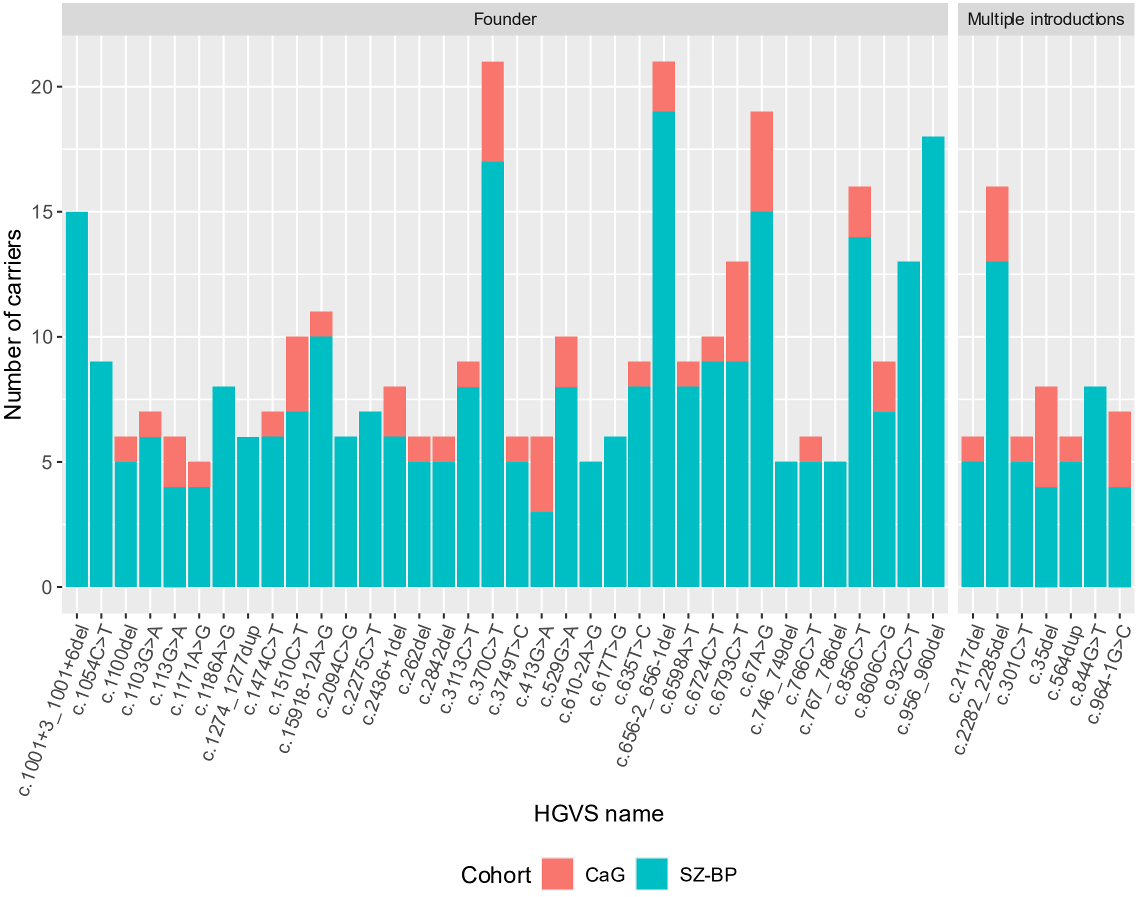


**Supplementary Figure S4.** Number of carriers in the Beauce cluster for each rare pathogenic variants with a relative frequency difference of at least 10% in Beauce compared to UrbanQc, categorized by cohort ($n_{CaG} = 69$, $n_{SZ\text{-}BP} = 248$). Variants are considered founder if at least 50% of the pairs of carriers are sharing IBD around the variant's position, else they were classified as variant with multiple introductions. (Red: CaG cohort, Blue: SZ-BP cohort)

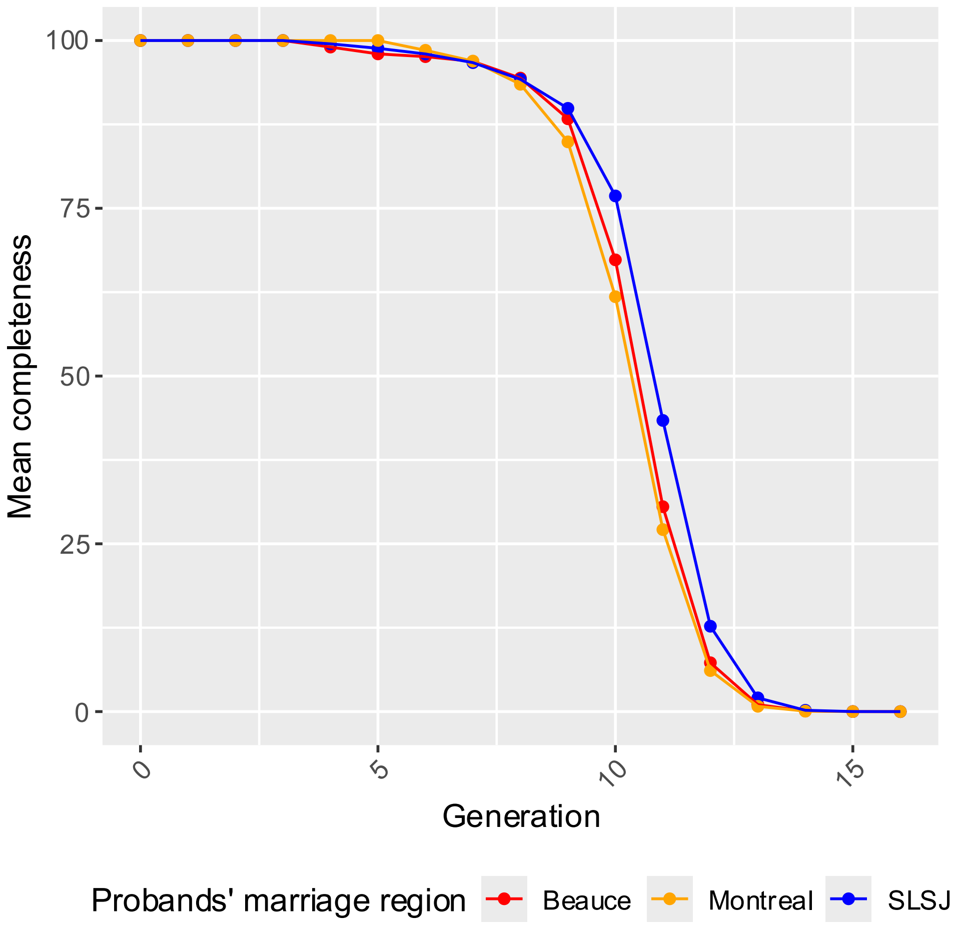


**Supplementary Figure S5.** Mean completeness per generation for each regional group (Red: Beauce, Orange: Montreal, Blue: Saguenay–Lac-St-Jean (SLSJ)**)**

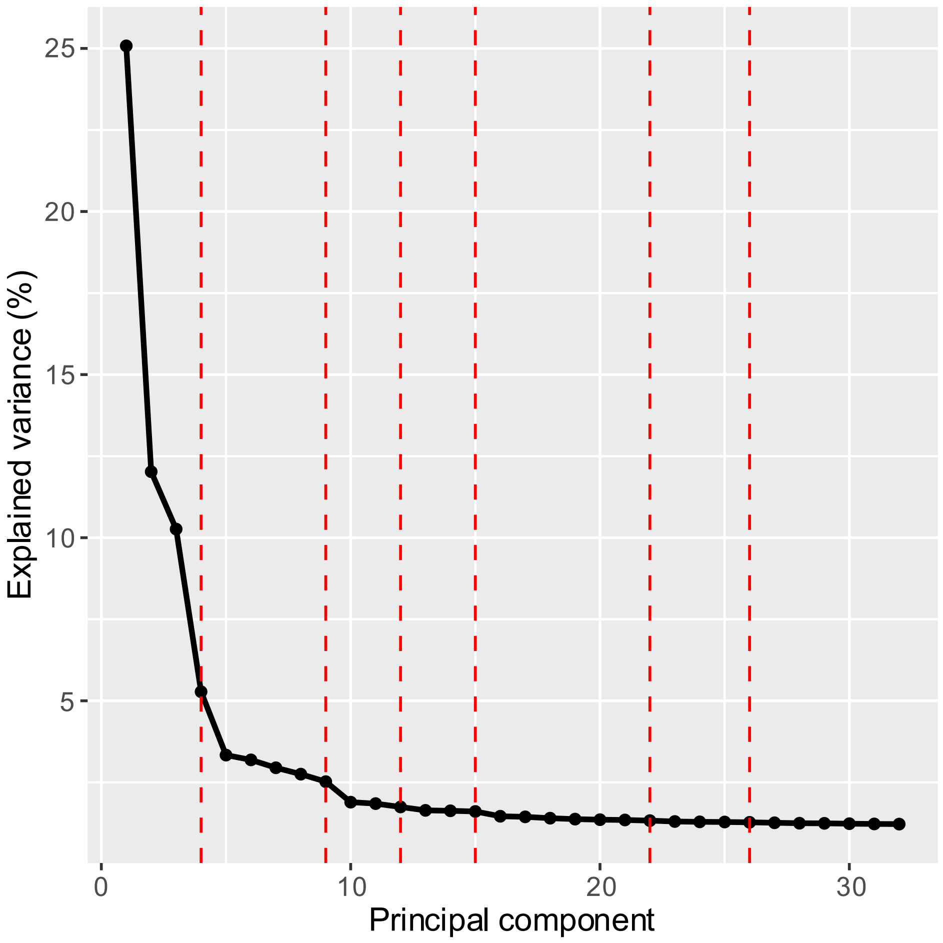


**Supplementary Figure S6**. Scree plot for the principal component analysis of the genotyping data. Red dotted lines represent the elbows identified using the change point detection method.

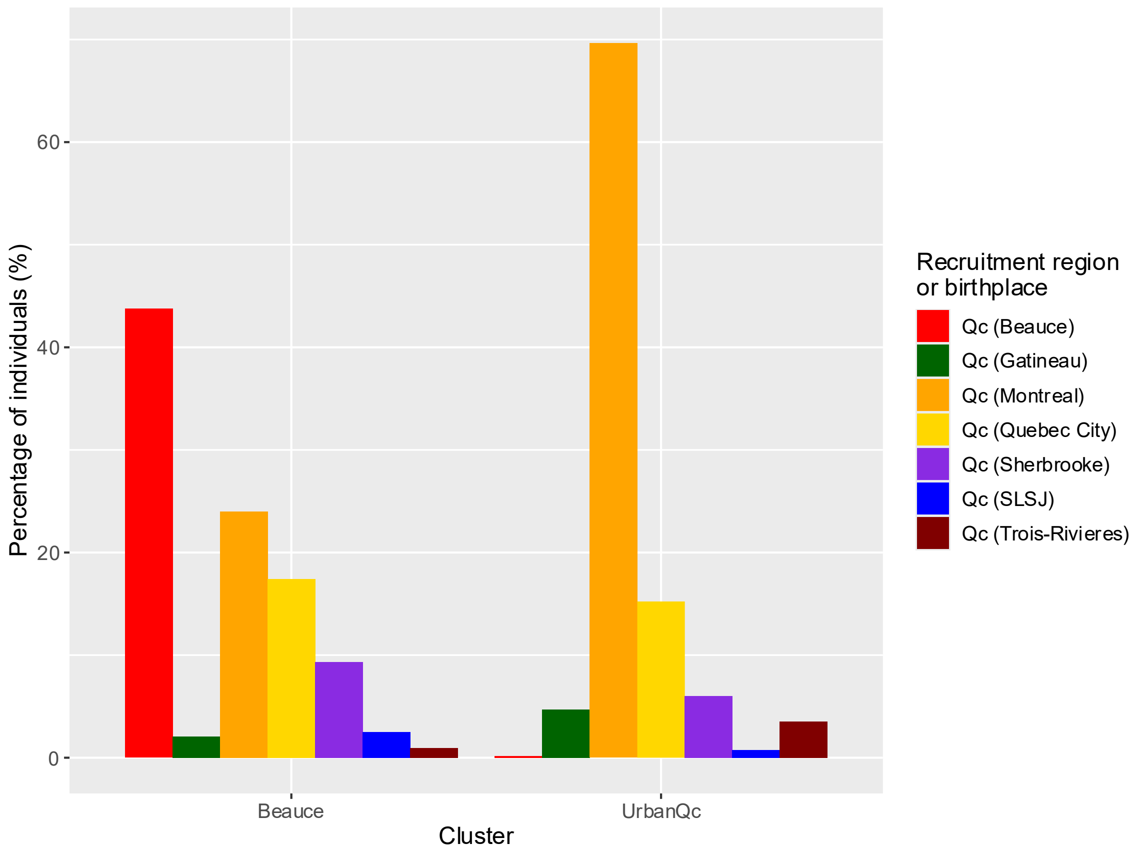


**Supplementary Figure S7.** Recruitment region or birthplace of individuals assigned to the Beauce and UrbanQc clusters as identified by DBSCAN on the UMAP derived from the 12 first principal components of the genotyping data.

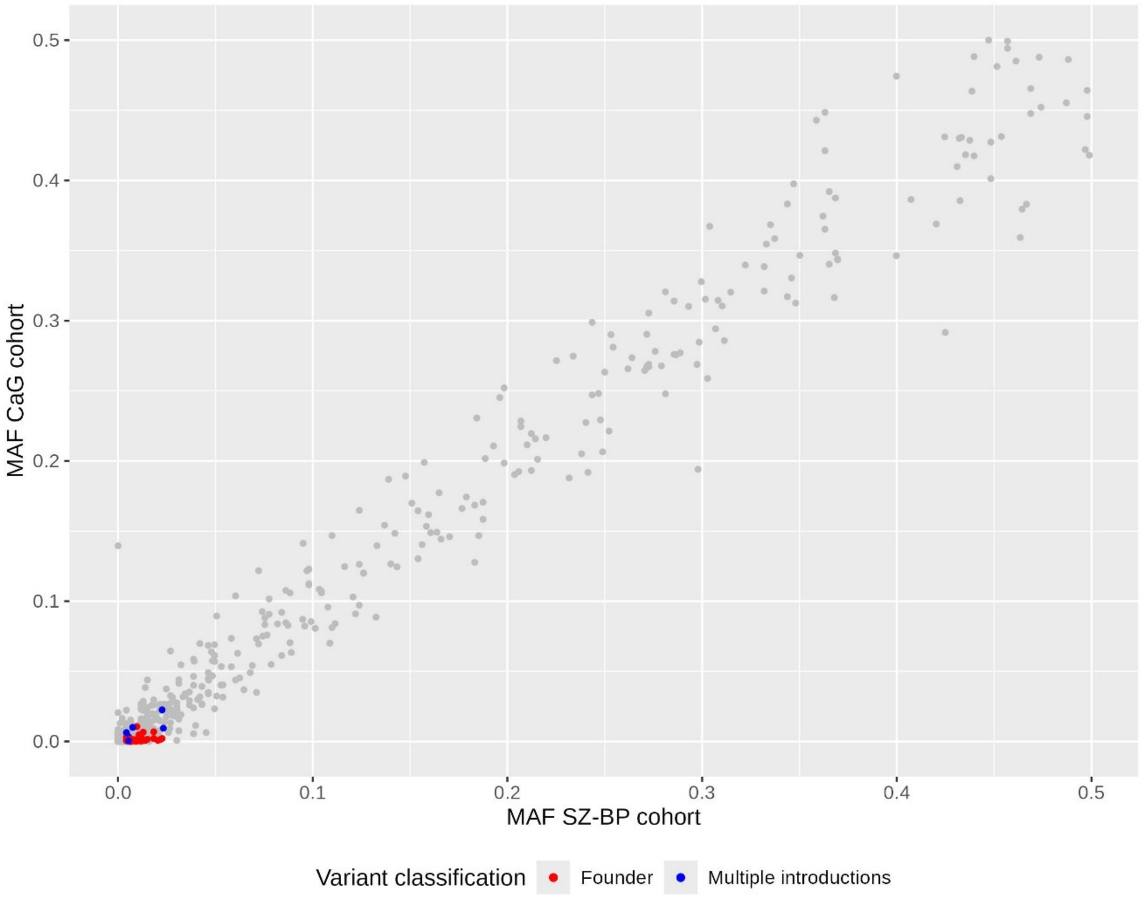


**Supplementary Figure S8.** Minor allele frequencies of the rare pathogenic variants in the SZ-BP cohort compared to the CaG cohort. Rare pathogenic variants with a relative frequency difference of at least 10% in Beauce compared to UrbanQc are highlighted (Red: founder variants, Blue: variants with multiple introductions).

**Supplementary references**


1. Chetaille, P. *et al.* Mutations in SGOL1 cause a novel cohesinopathy affecting heart and gut rhythm. *Nat. Genet.* **46**, 1245–1249 (2014).

2. Leveille, E. *et al.* Triple A syndrome presenting as complicated hereditary spastic paraplegia. *Mol. Genet. Genomic Med.* **6**, 1134–1139 (2018).

3. Cruz Marino, T. *et al.* Portrait of autosomal recessive diseases in the French-Canadian founder population of Saguenay-Lac-Saint-Jean. *Am. J. Med. Genet. A.* **191**, 1145–1163 (2023).

4. Michel, É. *et al.* Rare diseases load through the study of a regional population. *medRxiv* 2024.10. 29.24316346 (2024).

5. Gros-Louis, F. *et al.* Mutations in SYNE1 lead to a newly discovered form of autosomal recessive cerebellar ataxia. *Nat. Genet.* **39**, 80–85 (2007).

6. Hou, Y., McInnes, B., Hinek, A., Karpati, G. & Mahuran, D. A Pro504 → Ser Substitution in the β-Subunit of β-Hexosaminidase A Inhibits α-Subunit Hydrolysis of GM2Ganglioside, Resulting in Chronic Sandhoff Disease. *J. Biol. Chem.* **273**, 21386–21392 (1998).
7. La Piana, R. *et al.* Spastic Paraparesis and Marked Improvement of Leukoencephalopathy in Aicardi–Goutières Syndrome. *Neuropediatrics* **45**, 406–410 (2014).
8. Carter, K. C. *et al.* Mutation at the phenylalanine hydroxylase gene (PAH) and its use to document population genetic variation: the Quebec experience. *Eur. J. Hum. Genet. EJHG* **6**, 61–70 (1998).
9. Zaneveld, J. *et al.* Comprehensive analysis of patients with Stargardt macular dystrophy reveals new genotype-phenotype correlations and unexpected diagnostic revisions. *Genet Med* **17**, 262–70 (2015).
10. Mahdieh, N. *et al.* Genotype, phenotype and in silico pathogenicity analysis of HEXB mutations: Panel based sequencing for differential diagnosis of gangliosidosis. *Clin. Neurol. Neurosurg.* **167**, 43–53 (2018).

11. Fiskerstrand, T. et al. Mutations in ABHD12 Cause the Neurodegenerative Disease PHARC: An Inborn Error of Endocannabinoid Metabolism. Am. J. Hum. Genet. 87, 410–417 (2010).

12. Estiar, M. A. The Genetics of Hereditary Spastic Paraplegia. (2022).

13. Bchetnia, M. *et al.* Genetic burden linked to founder effects in Saguenay–Lac-Saint-Jean illustrates the importance of genetic screening test availability. *J. Med. Genet.* **58**, 653–665 (2021).

14. Scriver, C. R. Human Genetics: Lessons from Quebec Populations. *Annu. Rev. Genomics Hum. Genet.* **2**, 69–101 (2001).

15. Sillon, G., Allard, P., Drury, S., Rivière, J.-B. & De Bie, I. The incidence and carrier frequency of Tay-Sachs disease in the French-Canadian population of Quebec based on retrospective data from 24 years, 1992–2015. *J. Genet. Couns.* **29**, 1173–1185 (2020).

16. Fierheller, C. T., Alenezi, W. M. & Tonin, P. N. The Genetic Analyses of French Canadians of Quebec Facilitate the Characterization of New Cancer Predisposing Genes Implicated in Hereditary Breast and/or Ovarian Cancer Syndrome Families. *Cancers* **13**, 3406 (2021).

17. Bujakowska, K. M. et al. Targeted Exon Sequencing in Usher Syndrome Type I. Invest. Ophthalmol. Vis. Sci. 55, 8488–8496 (2014).

18. Gauthier, A., Wagner, E., Thibeault, R. & Lavoie, A. A Novel Case of Complement Factor B Deficiency. J. Clin. Immunol. 41, 277–279 (2021).

19. Moisan, L., Iannuzzi, D., Maranda, B., Campeau, P. M. & Mitchell, J. J. Clinical characteristics of patients from Quebec, Canada, with Morquio A syndrome: a longitudinal observational study. Orphanet J. Rare Dis. 15, 270 (2020).
