# Supplementary material for: Rare variants and founder effect in the Beauce region of Quebec
Source: Commun Biol. 2025 Aug 8;8:1184. doi: 10.1038/s42003-025-08630-7 (PMC12334588; doi:10.1038/s42003-025-08630-7)
Supplement: Supplementary file 4 — Description of Additional Supplementary Files [file 42003_2025_8630_MOESM4_ESM.docx]

Description of Additional Supplementary Files

**File name:** Supplementary Data 1

**Description:** Full description of the 43 rare pathogenic variants with a higher frequency in Beauce. The table includes gene and genomic position, disease and inheritance mode, minor allele frequency (MAF) in non-Finnish European (NFE), Beauce and UrbanQC, carrier rate (CR) in Beauce and UrbanQc, relative frequency difference between Beauce and UrbanQc, proportion of IBD sharing at the variant’s location, status of the variant and reference is the variant was previously reported in Quebec.

**File name:** Supplementary Data 2

**Description:** Minor allele frequency (MAF) and carrier rate (CR) for the 36 founder variants calculated on the whole Beauce and UrbanQc clusters as well as on a maximally unrelated subset.
