# Supplementary material for: Rare variants and founder effect in the Beauce region of Quebec
Source: Commun Biol. 2025 Aug 8;8:1184. doi: 10.1038/s42003-025-08630-7 (PMC12334588; doi:10.1038/s42003-025-08630-7)
Supplement: Supplementary file 5 — Reporting Summary [file 42003_2025_8630_MOESM5_ESM.pdf]

nature portfolio

Corresponding author(s): Simon Girard

Last updated by author(s): Jul 27, 2025

Reporting Summary

Nature Portfolio wishes to improve the reproducibility of the work that we publish. This form provides structure for consistency and transparency in reporting. For further information on Nature Portfolio policies, see our Editorial Policies and the Editorial Policy Checklist.

Statistics

For all statistical analyses, confirm that the following items are present in the figure legend, table legend, main text, or Methods section.

n/a	Confirmed	
☐	☒	The exact sample size (n) for each experimental group/condition, given as a discrete number and unit of measurement
☐	☒	A statement on whether measurements were taken from distinct samples or whether the same sample was measured repeatedly
☒	☐	The statistical test(s) used AND whether they are one- or two-sided<br>*Only common tests should be described solely by name; describe more complex techniques in the Methods section.*
☒	☐	A description of all covariates tested
☒	☐	A description of any assumptions or corrections, such as tests of normality and adjustment for multiple comparisons
☐	☒	A full description of the statistical parameters including central tendency (e.g. means) or other basic estimates (e.g. regression coefficient) AND variation (e.g. standard deviation) or associated estimates of uncertainty (e.g. confidence intervals)
☒	☐	For null hypothesis testing, the test statistic (e.g. F, t, r) with confidence intervals, effect sizes, degrees of freedom and P value noted<br>*Give P values as exact values whenever suitable.*
☒	☐	For Bayesian analysis, information on the choice of priors and Markov chain Monte Carlo settings
☒	☐	For hierarchical and complex designs, identification of the appropriate level for tests and full reporting of outcomes
☒	☐	Estimates of effect sizes (e.g. Cohen's d, Pearson's r), indicating how they were calculated

Our web collection on statistics for biologists contains articles on many of the points above.

Software and code

Policy information about availability of computer code

Data collection	No software were used during the data collection.
Data analysis	All genealogical analyses were performed using the python library GeneaKit version 0.1.0. The genetic data quality control was performed using plink/1.9b_5.2-x86_64. The principal component analysis was performed using the PC-AiR function from the R library GENESIS/2.28.0. The uniform manifold approximation and projection was performed with the R umap/0.2.10.0 library, and the clustering with dbscan/1.2.2. Identical-by-descent segments were inferred on the phased genotypes with refinedIBD version 17Jan2041 within Beagle version 18May20 and segments were merged using the merge-ibd-segments 17Jan20.102 tool. We inferred effective population size using the ibdne software (version 23Apr20.ae9)<br>All custom code is available on GitHub :https://github.com/Genopop/Rare_founder_variants_Beauce

For manuscripts utilizing custom algorithms or software that are central to the research but not yet described in published literature, software must be made available to editors and reviewers. We strongly encourage code deposition in a community repository (e.g. GitHub). See the Nature Portfolio guidelines for submitting code & software for further information.

Data

Policy information about availability of data

All manuscripts must include a data availability statement. This statement should provide the following information, where applicable:

- Accession codes, unique identifiers, or web links for publicly available datasets
- A description of any restrictions on data availability
- For clinical datasets or third party data, please ensure that the statement adheres to our policy

Access to the genealogical data used in this study may be granted to researchers for scientific purposes, provided that the request aligns with the objectives of the data file. Requests must be submitted to the BALSAC Project's Researcher Services, which is responsible for managing and evaluating access applications. To request access, researchers must complete the access request form available on the BALSAC website and send it to balsac@uqac.ca. Further information is available at: https://balsac.uqac.ca/en/acces-donnees/.
The aggregated data necessary to reproduce Figure 2 and 3 is available on GitHub (https://github.com/Genopop/Rare_founder_variants_Beauce). However, the source data and individual data points cannot be made available due to privacy concerns.
Due to recent data protection regulations in Quebec, the sharing of raw genomic data is subject to strict constraints. As a result, data from the Eastern Quebec schizophrenia and bipolar disorder kindred study is not publicly available at this time. Efforts are currently underway to deposit the data with the Centre québécois de données génomiques. In the meantime, data may be made available upon reasonable request from MM. The data from the CARTaGENE cohort is publicly available via an independent data access committee by the CARTaGENE cohort (https://cartagene.qc.ca/en/researchers/access-request.html). The source data behind Figure 4 is also available on GitHub.
All other data supporting the findings of this study are available from the corresponding author upon reasonable request, subject to ethical approvals and privacy considerations.

Research involving human participants, their data, or biological material

Policy information about studies with human participants or human data. See also policy information about sex, gender (identity/presentation), and sexual orientation and race, ethnicity and racism.

Reporting on sex and gender	No sex- or gender-based analyses were performed. For genealogical analyses, all individuals were grouped together, as our focus was on population-level trends rather than sex- or gender-specific patterns. Similarly, sex and gender were not considered in genetic analyses. Individuals were selected based on their regional origin, as determined by our ancestral clusters, irrespective of sex or gender. Additionally, the rare pathogenic variants analyzed were autosomal and not sex-linked.
Reporting on race, ethnicity, or other socially relevant groupings	This study was conducted among the population of french canadian descent with the aim of exploring genetic diversity within the regional populations.<br>For genealogical data, groups were formed based on the probands' first marriage region, and we ensured that it matched the first marriage region of their parents and grandparents.<br>For genetic analyses, the recruitment region and, for individuals born outside Canada, their country of birth were available. We only retained individuals who reported that they were born inside of Canada. Individuals were grouped into clusters, with ancestry inferred based on the predominant recruitment region or birth country within each cluster. We identified one cluster associated with Beauce ancestry, one with Saguenay–Lac-Saint-Jean ancestry and another comprising individuals from urban regions of Quebec.
Population characteristics	All individuals were recruited in the province of Quebec, and only those of French-Canadian descent from specific regions were included. Participants in the CARTaGENE cohort were recruited without regard to medical conditions, whereas the Eastern Quebec Schizophrenia and Bipolar Disorder Kindreds study cohort included individuals diagnosed with schizophrenia or bipolar disorder. Although various phenotypic descriptors were available for both cohorts, they were not used in this study.
Recruitment	Genealogies were reconstructed using Catholic marriage records and civil registry data from the BALSAC database. We included all individuals married in Beauce, Saguenay–Lac-Saint-Jean, and Montreal between 1935 and 1960.<br>Participants in the CARTaGENE are individuals aged between 40 and 69 who were recruited based on their residing regions (Montreal, Quebec City, Trois-Rivières, Sherbrooke, Gatineau, Saguenay).<br>Individuals in the Eastern Quebec Schizophrenia and Bipolar Disorder Kindreds study cohort were recruited in Beauce, Saguenay–Lac-Saint-Jean, and Îles-de-la-Madeleine following a schizophrenia or bipolar disorder diagnosis. Unaffected member of their families were recruited as well. Diagnosis was not considered in our analyses, and the selected rare variants in the study were not associated with specific conditions.
Ethics oversight	This study was approved by the Université du Québec à Chicoutimi (2024-1396) and the Centre Intégré Universitaire de Santé et de Service Sociaux de la Capitale-Nationale (2019-1597, NSM) ethics boards. Written informed consent was obtained from all adult participants or from the parents for participants under 18 years of age. All ethical regulations relevant to human research participants were followed.

Note that full information on the approval of the study protocol must also be provided in the manuscript.

Field-specific reporting

Please select the one below that is the best fit for your research. If you are not sure, read the appropriate sections before making your selection.

☒ Life sciences ☐ Behavioural & social sciences ☐ Ecological, evolutionary & environmental sciences

For a reference copy of the document with all sections, see nature.com/documents/nr-reporting-summary-flat.pdf

Life sciences study design

All studies must disclose on these points even when the disclosure is negative.

Sample size	Sample sizes were not pre-determined using power calculations; all available and eligible individuals were included to maximize representation.The sample size for the genealogical analyses represents the maximum available data that passed quality control for each regional group. For the bootstrapped analysis, the replicated sample size corresponds to the sample size of the smallest regional group.<br>For the genetic analyses, we used data from existing cohorts and retained only individuals of Beauce ancestry or from the urban region of Quebec, based on our ancestry-based clustering. Given that the present-day Beauce population results from a regional founder effect, we believe fewer samples are necessary to accurately represent this population compared to more admixed regions.
Data exclusions	For the genealogical analyses, we only retained probands for whom the two parents and four grand-parents came from specific regional groups and the genealogies were of sufficient completeness. We also excluded probands presenting relations of the first degree.<br>Genetic samples were excluded if they did not pass the standard quality control, if they reported being born outside of Canada or, later in the analyses, if they were not comprised in the clusters of relevance to our study.
Replication	The genealogical data included all available data for our three regional groups at the time of the study. We compared our results with those of previous studies where possible and were able to confirm their reliability. For the genetic analyses, replication of our findings was not possible since we used all the available whole genome sequencing data for individuals of Beauce ancestry. However, we followed a methodology demonstrated to be reproducible in a previous study and successfully confirmed some of our identified founder variants.
Randomization	For the genealogical analyses, groups were formed based on the marriage region of the probands. To assess the ancestor diversity ratio, random groups of 7,445 probands were formed with the possibility of resampling. This process was repeated 1,000 times to ensure comprehensive coverage of the entire samples.<br>For the identification of founder variants, groups were formed using a uniform manifold approximation and projection performed on the 12 first principal components (PC) of a principal component analysis (PCA) based on the genotyping data. To ensure that the PCA was not biaised by the presence of relatedness in the cohort, we computed ancestry-based PC on a subset of unrelated individuals (up to the third degree), then projected the related individuals using the PC-AiR function from the GENESIS library. Individuals were clustered using density-based spatial clustering of applications with noise. This allowed us to select individuals with shared regional ancestry and facilitated the identification of rare variants that reached higher frequencies due to the founder effect.
Blinding	Blinding was not deemed necessary for the purpose of our study as our goal was to describe the regional population of Beauce and to identify rare pathogenic variants that are more frequent in this region due to the founder effect.

Reporting for specific materials, systems and methods

We require information from authors about some types of materials, experimental systems and methods used in many studies. Here, indicate whether each material, system or method listed is relevant to your study. If you are not sure if a list item applies to your research, read the appropriate section before selecting a response.

Materials & experimental systems

n/a	Involved in the study	
☒	☐	Antibodies
☒	☐	Eukaryotic cell lines
☒	☐	Palaeontology and archaeology
☒	☐	Animals and other organisms
☒	☐	Clinical data
☒	☐	Dual use research of concern
☒	☐	Plants

Methods

n/a	Involved in the study	
☒	☐	ChIP-seq
☒	☐	Flow cytometry
☒	☐	MRI-based neuroimaging

Plants

Seed stocks	*Report on the source of all seed stocks or other plant material used. If applicable, state the seed stock centre and catalogue number. If plant specimens were collected from the field, describe the collection location, date and sampling procedures.*
Novel plant genotypes	*Describe the methods by which all novel plant genotypes were produced. This includes those generated by transgenic approaches, gene editing, chemical/radiation-based mutagenesis and hybridization. For transgenic lines, describe the transformation method, the number of independent lines analyzed and the generation upon which experiments were performed. For gene-edited lines, describe the editor used, the endogenous sequence targeted for editing, the targeting guide RNA sequence (if applicable) and how the editor was applied.*
Authentication	*Describe any authentication procedures for each seed stock used or novel genotype generated. Describe any experiments used to assess the effect of a mutation and, where applicable, how potential secondary effects (e.g. second site T-DNA insertions, mosiacism, off-target gene editing) were examined.*
